# Mid-term results of cemented hip arthroplasties through the direct anterior approach in the lateral decubitus position: a retrospective cohort study

**DOI:** 10.1186/s13018-024-04696-x

**Published:** 2024-04-01

**Authors:** Wietse P.R. Melman, Harmen B. Ettema, Mireille A. Edens, Cees C.P.M. Verheyen

**Affiliations:** 1 Department of Orthopedic Surgery and Traumatology, Isala Hospital, Zwolle, The Netherlands; 2 Epidemiology Unit, Department Innovation and Science, Isala Hospital, Zwolle, The Netherlands; 3 Department of Orthopedic Surgery and Traumatology, Saxenburgh Medisch Centrum, Jan Weitkamplaan 4a, Hardenberg, 7772 SE The Netherlands

**Keywords:** Hip, Anterior, Lateral decubitus, Surgical approach, Complications, Learning curve

## Abstract

**Background:**

Reports show a high complication rate when starting with the Direct Anterior Approach (DAA) in a supine position for hip arthroplasty. The DAA with the patient in lateral decubitus position may avoid this problem because it supposedly provides better visibility, especially on the femoral side. However, this approach did show a rather high complication rate during the adoption of the approach at 1 year follow up in our previous report. We were interested what the overall 7 year survival estimate would be and whether improvement could be seen with growing experience.

**Methods:**

A cohort of patients undergoing total hip arthroplasty right from the start of applying the DAA in lateral decubitus position was analysed.

**Results:**

In total 175 hip prostheses (162 patients) were evaluated. The 7-year survival estimate was 95.1%, 95 CI: 91.8–98.4%. In 6 of 8 revisions there was aseptic loosening of the stem. By dividing the cohort into 3 consecutive groups in time we did not see a significantly improving revision rate.

**Conclusions:**

In our experience, the adoption of the direct anterior approach in lateral decubitus position caused a relatively low 7-year survival estimate without an apparent decrease with growing experience, however given the low number of cases further research is needed to investigate the long-term risk of adopting a new approach.

## Background

The direct anterior approach (DAA) uses a small skin incision (8–10 cm) and follows a true intermuscular, intervascular and interneural plain to expose the hip joint. Reports claim the DAA has multiple operative and early postoperative benefits compared to other approaches [1–6]. The evidence however is of limited quality and with conflicting outcomes [7]. Moreover, the benefit of minimal invasive hip arthroplasty in general is debatable [8]. Also, there is only limited literature on mid-to-long-term results of DAA hip surgery performed in the learning curve and thus the risk of adopting this approach [9].

In the vast majority of studies describing the anterior approach, the patient is positioned supine with or without traction [1, 10–22]. This approach can also be carried out in the lateral decubitus position [23]. A theoretical advantage of this position is that it causes less effort to expose the femur during surgery while the leg is simply positioned in hyperextension, abduction and external rotation. This position provides easier access to the femoral canal without inducing possible complications when traction is applied.

In 2015 we published our first experiences with the DAA in lateral decubitus position for primary hip arthroplasty. The primary aim of that study was to examine the learning curve, with a follow up of 1 year. Secondary outcomes being observed were overall, peri-operative and post-operative complication rates as well as revision rates up to 1 year after surgery [24]. We now present the 7-year survival of this cohort and again investigated if a learning curve effect was present.

## Methods

We retrospectively analysed the learning curve for one surgeon adopting the DAA in lateral decubitus position for primary hip arthroplasty. The surgeon (CCPMV) had carried out or strictly supervised all operations. The surgeon had no previous surgical experience with this approach on patients but had performed over 1000 hip arthroplasties using the anterolateral abductor split approach in the lateral decubitus position. Before the first procedure, a human cadaver dissection course was followed and the first 4 procedures were supervised by a recognized expert on this approach. The study was conducted in the Isala hospital, Zwolle, The Netherlands. Patients operated between 2009 and 2013 were analysed.

### Operative technique and perioperative care

The operation was carried out according to the technique described by Michel and Witschger [23]. They describe an anterior, minimally invasive, surgical approach to the hip joint in lateral decubitus position on a regular operation table without traction. A longitudinal skin incision is used. Thereafter the hip joint is approached between both tensor fasciae latae and gluteus medius muscle (lateral) and sartorius and rectus femoris muscle (medial). No tendons or muscles are cut or detached. The osteotomy of the femoral neck is performed in situ and subsequently the head is removed. To access the femoral canal, the operated leg is placed in hyperextension, abduction and external rotation posteriorly. Cemented F.A.L.® (Link, Hamburg, Germany) acetabular components and cemented Lubinus SP-II® (Link, Hamburg, Germany) stems were implanted in all our patients. The preparation of the femur and acetabulum was performed using angled handles for the acetabular reamers and offset femoral broaches. PALACOS® R + G cement (Heraeus, Hanau, Germany) was used with a 3rd generation cementing technique. BIOLOX® forte ceramic 28 mm femoral heads were used (CeramTec GmbH, Plochingen, Germany). No intra-operative fluoroscopy was used.

Mobilization was started the first day after surgery under the supervision of a physiotherapist. The local rapid recovery protocol at the time aimed for a 3- to 4-day hospital stay. Patients received antithrombotic prophylaxis (fondaparinux 2.5 mg once daily s.c.) up to 6 weeks postoperatively. Radiological and clinical evaluations were conducted 6 weeks, 1 year, 3 years and 5 years after the index operation.

### Participants

All patients receiving primary total hip arthroplasty using a DAA with at least 7 years of follow-up were included. Not all primary hip cases in the analysed period were operated using the DAA. The applied surgical approach was left at the surgeon’s discretion, i.e. either the new direct anterior or familiar anterolateral abductor split approach. In this way the surgeon could resort to his regular approach, either in cases subjectively assessed as difficult (local large fat deposits or muscle hypertrophy), or because of logistic reasons (unavailability of angled reamers). This was discussed with patients in the outpatient clinic but ultimately decided in the operating theatre.

### Variables

The primary end point was the 7-year survival estimate of the hip arthroplasties. Also, we looked for a potential learning effect with growing experience.

Revision status, reasons for revision, demographic and perioperative data were retrieved from the medical record and, if missing, by calling patients.

The anteversion and inclination of the cup, as well as the leg length change on the post operative X-ray were analysed. Control criteria performed on the radiographs were symmetrical imaging of the pelvis (anteversion) and collum femoris and the trochanter minor clearly visible on the medial side of the femur (leg discrepancy) [25]. Leg lengthening/shortening was measured using pre-operative and post-operative radiographs. The acetabular teardrop was chosen as a landmark on the pelvis and the most medial part of the lesser trochanter on the femoral side. This method has been reported to be as reliable as orthoroentgenograms and reproducible [26–29]. Leg lengthening was considered a suboptimal result given this is associated with an inferior range of motion [27]. Cup inclination and anteversion were measured according to the method validated and described by Lu et al. [30]. A cup position of 30–55 degrees abduction and 0–10 anteversion was aimed for. Further peri-operative outcomes measures were reported in our previous publication about this cohort with 1 year follow up [24].

We performed a Kaplan-Meier analysis for the 7 years survival estimate of the hip arthroplasties in the cohort, deceased patients were censored to the right.

In order to identify a learning effects, the cohort was divided into 3 consecutively admitted groups. The first group contained the first 59 procedures, the second the following 58 and the third the last 58 procedures. We chose 3 groups of about equal group size because earlier research indicated that the learning curve for the DAA takes around 46 operations [15]. We calculated revision percentages for the 3 consecutive groups with 95% confidence intervals (Wilson procedure with continuity correction) to look for a potential learning effect. The presence of a normal distribution of continuous data was visually assessed based on Q-Q plots. All analyses were performed using SPSS 20 for Windows (SPSS Inc., Chicago, IL, USA).

## Results

In total the surgeon implanted 230 total hip prostheses between October 2009 and April 2013 for primary hip osteoarthritis. 48 cases were excluded because the surgeon relied on his familiar anterolateral abductor split approach. When the first third of the DAA cohort was operated the surgeon resorted to the anterolateral abductor split approach in 36 cases, during the middle third in 10 cases and in the final third in 2. In total 175 cemented hip prostheses were implanted in 162 patients via the direct anterior approach for primary hip osteoarthritis and subsequently analysed.

7 patients had a follow up between 1 and 7 years, at that time no complications were recorded. These cases were included in the analysis as not revised. 10 patients died during follow. Demographic data of the total cohort is listed in Table 1. The demographic data for the different groups were compared and showed no significant differences.

**Table 1:** Demographic data

Patients / hip replacements	162/175
Male, % Female, %	23 77
Mean age during operation (years; STD)	70 (7.5)
Mean BMI (STD)	28 (4.5)
ASA, % 1 2 3 4	23 71 5 1
Preoperative diagnosis, % Primary osteoarthritis	100

Overall operative outcome is summarized in Table 2. The mean cup inclination was 49°, 83% fell within the range of the targeted 30°-55° inclination, no cup had less than 35° inclination. Fourteen radiographs were rejected for anteversion measurements and nineteen for leg length measurements.

**Table 2:** Overall operative outcome

Mean leg length increase/decrease (mm; ±STD)	1; ±7
Mean cup anteversion (degrees; ±STD)	13; ±6
Mean inclination cup, (degrees; ±STD)	49; ±6

**Fig. 1:**
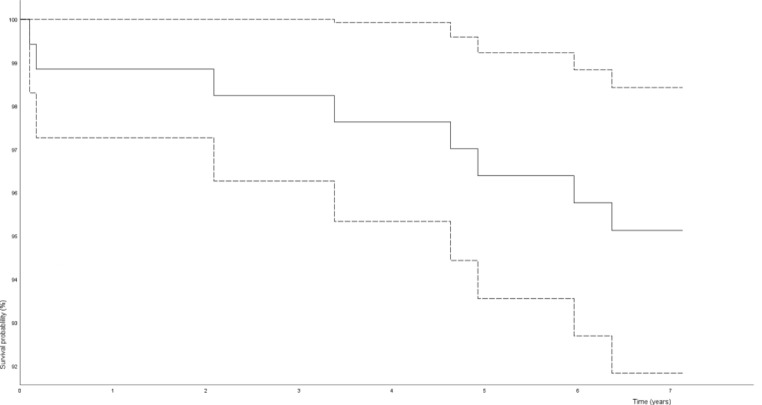
Survival curve for the THA, dotted lines show the 95% confidence interval

No evident learning curve effect was observed for the revision rate at 7 years follow up (Table 3). Revision surgery within 7 years was indicated in 1 case for early infection, in another for recurrent dislocations and in 6 cases for aseptic loosening, in 4 of these the stem and cup were loose and revised and in 2 only the stem. Revision surgeries and their cause are also summarized in Table 4. The overall 7-year survival estimate in our Kaplan-Meier analyses was 95.1%, 95% CI: 91.8–98.4% (Fig. 1).

**Table 3:** Learning effect DAA

	Group 1 (*n* = 59)	Group 2 (*n* = 58)	Group 3 (*n* = 58)
Revisions rate at seven-year follow-up % (95 C.I.)	5.1 (1.3–15.1)	3.4 (0.6–13.0)	5.2 (1.3–15.3)

**Table 4:** Revisions and cause

Revision number	Time to revision	Type of revision	Reason for revision
1	1 month	Cup and stem, after failed dair	Infection. Initial DAIR surgery was performed because of prolonged wound leakage.
2	2 months	Cup and stem	Instability. 3 early hip dislocations. CT showed malposition of the stem with ~ 45 degrees of anteversion.
3	2 years	Cup and stem	Aseptic loosening. Initial placement of the stem in varus and steep placement of the cup at 60 degrees.
4	3 years	Stem	Aseptic loosening. Initial proximal anterior placement of the stem with the distal tip placed against the posterior cortex.
5	4 years	Cup and stem	Aseptic loosening. Initial good placement on the post operative radiograph.
6	4 years	Stem	Aseptic loosening. Initial varus placement of the stem.
7	5 years	Cup and stem	Aseptic Loosening. Initial good placement on the post operative radiograph.
8	6 years	Cup and stem	Aseptic loosening of the cup and stem. Initial post operative radiographs showed varus placement of the stem and lucency cranial of the cup.

## Discussion

The study revealed that our adoption of the DAA in lateral decubitus position resulted in a relatively low 7-year survival estimate of (95.1 ). We did not see the revision rate improve with growing experience in our cohort of 175 procedures. We believe our results are supported by a good follow up rate.

There is an ongoing debate about which surgical approach to use for total hip arthroplasty. Speaking against the DAA is the relatively high revision rate we saw which is also reported in various articles. A recently published retrospective study compared different surgical approaches and found the complication rate of the posterior approach to be significantly lower than the anterior approach, 5.9% (97/1657) vs. 8.5% (113/1329; *p* = 0.006) at a mean follow-up of 3.7 years [31]. All operations in the anterior approach group were performed by surgeons that adapted the approach well before the start of the study. This suggests that even beyond the learning curve, the complication rate remains relatively high. Furthermore, literature with longer follow-up also shows similar high revision rates. Gofton et al. found a revision rate of 5.5% at 5 years analysing 364 cases operated by 4 different surgeons with variable experience [32]. Furthermore, concerning the learning curve, an Australian registry study showed a revision rate of 6% at 5 years follow up for the first 15 operations performed and 3% for the subsequent 15 operations [33]. Other studies however show similar low revision rates comparing the DAA with the posterior approach, for instance the one from Angerame et al. with 5 years follow up [34].

Early femoral failure may be a cause of this relatively high revision rate in patients operated using the anterior approach. A retrospective study that analysed 478 revisions performed within 5 years after the primary surgery found the anterior approach to be a significant predictor of early femoral failure. In patients who had initially undergone the direct anterior approach femoral failure was present in 50.9% of the revisions, for the direct lateral approach this was 35% and the posterior approach 14% [35]. Another study using the Dutch Arthroplasty Registry found the anterior approach to be associated with a higher risk of revision for stem loosening at 5 years follow up compared to the posterolateral approach [36]. Furthermore, our study also identified the stem as a weak link. In all our revisions for aseptic loosening the stem was involved. The lateral decubitus position may also play a role in this, Chen et al. showed this by comparing 76 cases of fluoroscopy-guided DAA arthroplasty, 38 in supine position and 38 in lateral decubitus. They concluded that the lateral decubitus position resulted in less favorable positioning of the femoral components compared to the supine position [37]. On the contrary, Rahm et al. retrospectively analysed 275 hip replacements using the DAA in supine positions and reported excellent 10-year survivorship (96,8%). However, the generalizability of this study may be limited since only 50% of all hip replacements performed during the time period were analysed, possibly excluding more difficult cases. Also, most arthroplasties were performed by a surgeon with a lot of experience in the direct anterior approach (38).

Another explanation for the relatively high revision rate in our study could be the relatively high average BMI (mean of 28) of the patients, since a high BMI seems to negatively impact the outcome of minimal invasive hip arthroplasty [38].

Overall cup inclination was relatively steep with 16% of procedures resulting in an inclination of more than 55°, outside the historical target values. This may not be a big problem since hip instability is likely multifactorial and the ideal cup position may be outside the Lewinnek safe zone [39]. Furthermore, only in one patient, with femoral component malplacement on CT, revision was needed because of multiple dislocations.

This study has some weaknesses however, firstly: the surgeon opted for his familiar abductor split approach in several cases, especially during the first group of the cohort. Therefore, more challenging cases were probably not yet operated, this selection bias may have shielded a possible learning effect. Secondly, the study may also be underpowered to adequately show a learning effect. Thirdly, the surgical approach is only one of the many factors affecting the revision rate. Another factor for instance is the implant that is used. Even though we used implants with a current ODEP rating of 15 A, these particular implants still may prove to be troublesome in our DAA approach. Also, because many factors influence longer term revision rate, using the revision rate as an outcome measure for assessing the learning curve is questionable. Finally, 7 cases had no follow up of 7 years, these were included in the analyses as not revised, possibly underestimating the revision rate.

Due to a perceived high initial complication rate the surgeon chose to stop using all DAA approaches for hip replacement surgery shortly after the studied period.

## Conclusions

There is only limited literature available about starting with the DAA and the risk this poses for the long-term survival of the implants. In our experience, the adoption of the direct anterior approach in lateral decubitus position caused a relatively low 7 years survival estimate. Furthermore, we did not see the revision rate improve with growing experience. Further research is needed to investigate the long-term risk of adopting a new approach.

## Data Availability

The datasets used and/or analysed during the current study are available from the corresponding author on reasonable request.
